# Forecasting Weekly Dengue Cases by Integrating Google Earth Engine-Based Risk Predictor Generation and Google Colab-Based Deep Learning Modeling in Fortaleza and the Federal District, Brazil

**DOI:** 10.3390/ijerph192013555

**Published:** 2022-10-19

**Authors:** Zhichao Li

**Affiliations:** Key Laboratory of Land Surface Pattern and Simulation, Institute of Geographic Sciences and Natural Resources Research, Chinese Academy of Sciences, Beijing 100101, China; lizc@igsnrr.ac.cn

**Keywords:** dengue risk prediction, big geospatial data, Google Earth Engine, cloud deep learning, Google Colab

## Abstract

Efficient and accurate dengue risk prediction is an important basis for dengue prevention and control, which faces challenges, such as downloading and processing multi-source data to generate risk predictors and consuming significant time and computational resources to train and validate models locally. In this context, this study proposed a framework for dengue risk prediction by integrating big geospatial data cloud computing based on Google Earth Engine (GEE) platform and artificial intelligence modeling on the Google Colab platform. It enables defining the epidemiological calendar, delineating the predominant area of dengue transmission in cities, generating the data of risk predictors, and defining multi-date ahead prediction scenarios. We implemented the experiments based on weekly dengue cases during 2013–2020 in the Federal District and Fortaleza, Brazil to evaluate the performance of the proposed framework. Four predictors were considered, including total rainfall (R_sum_), mean temperature (T_mean_), mean relative humidity (RH_mean_), and mean normalized difference vegetation index (NDVI_mean_). Three models (i.e., random forest (RF), long-short term memory (LSTM), and LSTM with attention mechanism (LSTM-ATT)), and two modeling scenarios (i.e., modeling with or without dengue cases) were set to implement 1- to 4-week ahead predictions. A total of 24 models were built, and the results showed in general that LSTM and LSTM-ATT models outperformed RF models; modeling could benefit from using historical dengue cases as one of the predictors, and it makes the predicted curve fluctuation more stable compared with that only using climate and environmental factors; attention mechanism could further improve the performance of LSTM models. This study provides implications for future dengue risk prediction in terms of the effectiveness of GEE-based big geospatial data processing for risk predictor generation and Google Colab-based risk modeling and presents the benefits of using historical dengue data as one of the input features and the attention mechanism for LSTM modeling.

## 1. Introduction

Dengue fever, one of the mosquito-borne diseases, is mainly distributed in tropical and subtropical urban and semi-urban areas worldwide [1,2]. Mosquito prevention and control is still the main measure for dengue epidemic prevention due to the lack of dengue vaccines. Dengue risk prediction is an important basis that permits providing valuable information for mosquito control decision-making. Due to global climate change, urbanization, and urban population growth, the need for efficient, accurate, and timely dengue risk prediction is even more urgent [3,4].

Different climate factors derived from weather stations, such as precipitation, temperature, and relative humidity, have been used in urban dengue risk prediction as they affect the life cycles, survival rates, and biting rates of *Aedes* mosquitoes, as well as the virus incubation period, thereby affecting the spatio-temporal patterns of dengue epidemics [5,6,7,8]. However, weak spatial representation of weather stations and massive data downloading and analysis confront dengue risk prediction with challenges [9]. In addition, vegetation indices, such as the normalized difference vegetation index (NDVI) and enhanced vegetation index (EVI) [10,11,12], have been used in dengue risk prediction as they could be regarded as a proxy of vector presence [13]; however, the time-consuming downloading and processing of satellite images and ready-to-use datasets to compute vegetation indices also make dengue risk forecasting a challenge. In this case, it is crucial to propose an efficient method for facilitating the generation of dengue risk predictors.

The advancement of geospatial data cloud computing platforms facilitates the identification of dengue risk predictors. For example, Google Earth Engine (GEE) (Mountain View, CA, USA) hosts multi-petabyte satellite images (e.g., MODIS, Landsat and Sentinel) and global scale ready-to-use datasets on different topics (e.g., climate, land cover, cropland, urbanization and population), provides different algorithms for image preprocessing, spatial and temporal analysis, and image classification, and supports parallel computation [14,15,16,17], which provides unprecedented opportunities for effectively generating data of dengue risk predictors. Moreover, it can accept the upload of external geographic information system (GIS) vector data for targeting specific study areas that permits generating the data of risk predictors according to the disease data, often collected from administrative unit-based statistics [16,17]. It is thus clear that depending on the GEE platform, selecting appropriate geospatial data and identifying the risk predictors according to the epidemic area and disease data are key issues for dengue risk prediction.

Diverse artificial intelligence approaches have been used in dengue risk prediction that include, but are not limited to, generalized additive model (GAM) [18,19,20], random forest (RF) [10,21,22], support vector machine (SVM) [23], artificial neural networks (ANNs) [6,10,19], and long-short-term memory (LSTM) [9,24,25,26]. Among them, LSTM is proposed to automatically identify the characteristics of long-term trends and short-term fluctuations of time series and become popular in dengue risk prediction. Moreover, it often couples with other mechanisms to simplify the dengue risk prediction or enhance the model accuracy in real-world applications. For example, LSTM with transfer learning enables the transfer of pre-trained LSTM features from one study area to another if the two areas have comparable climate and environmental conditions and dengue epidemics [24]. LSTM with attention mechanisms (namely LSTM-ATT) adds an attention layer after LSTM architecture and can assign a weight for each hidden state, which attends to the different sequence steps for improving the power of exploiting information [26]. In addition, the parameter time step of the LSTM model, which is the length of the input time series, makes it possible to explain the effect of climate and environmental conditions on dengue risk in the past [9,24]. Deep learning approaches also play important roles in the prediction of other infectious diseases, such as the use of the neural network for weekly Zika risk prediction [27] and the use of the gated recurrent unit (GRU) for predicting the weekly influenza cases at both the city-level and state-level [28]. Despite these successful applications of deep learning models in infectious disease risk prediction, model training is still a major issue that is time-consuming and very computationally intensive.

Cloud deep learning is a good choice to accelerate model training with distributed hardware, and dengue risk prediction can benefit greatly from computing resources. For example, Google Colab (Mountain View, CA, USA), a free web platform with free resources of the Google servers, provides a serverless Jupyter notebook for interactive development, supports deep learning frameworks, and enables model training and evaluation using Tensorflow [29,30]. To date, the applications of Google Colab in public health are still limited, which has not been used in dengue risk prediction yet.

In this context, this study aims to propose a framework by integrating GEE and Google Colab for efficient and accurate city-level dengue risk prediction. Specifically, it expects to effectively generate the dengue risk predictors using big geospatial data cloud computing based on the GEE platform, and accurately predict dengue risk based on different artificial intelligence models on Google Colab. This study expects to show the potential of integrating GEE and Google Colab in public health.

## 2. The Framework for Dengue Risk Prediction Based on GEE and Google Colab

**Figure 1:**
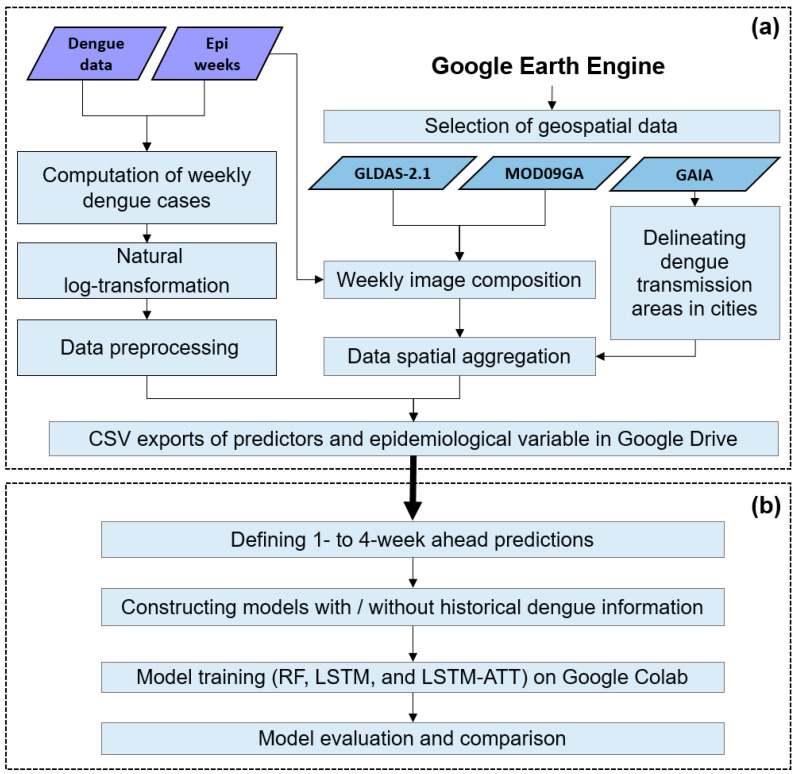
The proposed framework for city-level dengue risk prediction by integrating the GEE platform and Google Colab. Part (**a**) shows the steps of generating risk predictors using big geospatial data cloud computing based on the GEE platform. Part (**b**) represents the steps of defining multi-week ahead forecast scenarios, model construction, training, and evaluation using Google Colab.

A framework of efficient and accurate city-level dengue risk prediction is proposed, which includes two parts (Figure 1). Part (a) represents the steps of generating risk predictors using big geospatial data cloud computing hosted on the GEE platform. Part (b) represents the steps of defining multi-date ahead forecast scenarios, model construction, training, and evaluation using Google Colab.

Part (a) includes: (1) defining the epidemiological weeks according to an epidemiological calendar and generating the dengue-dependent factor by counting the number of dengue cases per week and naturally log-transformed weekly dengue case plus 1 to obtain a more stationary dependent factor; (2) considering epidemiological week and generating a suite of weekly image composites by stacking sub-daily or daily images between the beginning date and the end date of epidemiological weeks and computing a value per pixel using an algorithm (e.g., minimum, maximum, mean or sum); (3) delineating the main area of dengue transmission by creating a buffer zone of 1 km around the impervious surface area and generating a time series for each predictor by spatially aggregating the values of pixels covering the buffer zone. Here, the maximum flight range of dengue vectors (i.e., 1 km for *Aedes aegypti* and *Aedes albopictus*) is considered as the distance of the buffer zone [31,32,33]. We assume that the majority of human activities are within the impervious surface area and *Aedes* mosquitoes prefer to live near people; (4) combining the time series of risk predictors and dengue epidemiological variable to generate a dataset in CSV format that is saved in Google Drive (Figure 1a).

Part (b) includes (1) defining multi-week ahead prediction scenarios by considering time lags in advance of dengue epidemics (i.e., 1- to 4-week ahead predictions); (2) constructing different artificial intelligence models by taking historical dengue time series as one of the input features or not; (3) training models and evaluating the performance of models by computing evaluation indices and model comparison (Figure 1b).

### 2.1. Models

Two deep learning models (i.e., LSTM and LSTN-ATT) were considered in this study to predict the dengue risk. In addition, we used a classical machine learning model (i.e., RF) as a baseline model to provide a comparison for understanding the performance of LSTM and LSTM-ATT.

#### 2.1.1. LSTM

The conventional recurrent neural network (RNN) is susceptible to gradient vanishing and gradient explosion and is incapable of learning nonlinear relationships from long sequences [34]. LSTM is proposed to automatically identify the characteristics of long-term trends and short-term fluctuations of time series. A typical LSTM cell has three gates: an input gate, an output gate, and a forget gate. The forget gate ($f_{t}$) uses the sigmoid activation function to selectively forget the irrelevant information [34]:

(1) $$f_{t}=\sigma \left(W_{f}\cdot \left[h_{t-1},x_{t}\right]+b_{f}\right)$$

where σ denotes the sigmoid function, $W_{f}$ denotes the weight matrix of the unit, $h_{t-1}$ denotes the hidden state at the previous time t−1, and $b_{f}$ denotes the bias parameter of the unit.

The input gate ($i_{t}$) decides what to memorize using the sigmoid and tanh functions:

(2) $$i_{t}=\sigma \left(W_{i}\cdot \left[h_{t-1},\;x_{t}\right]+b_{i}\right)$$

(3) $$\hat{C_{t}}=\tanh\left(W_{C}\cdot \left[h_{t-1},x_{t}\right]+b_{C}\right)$$

(4) $$C_{t}=f_{t}*C_{t-1}+i_{t}*\hat{C_{t}}$$

where $W_{i}$ and $b_{i}$ represent the weight matrix and bias of the unit, respectively, $C_{t}$ denotes the state of the memory unit.

The output gate ($o_{t}$) determines the exact output from the current cell and updates the historical state:

(5) $$o_{t}=\sigma \left(W_{o}\cdot \left[h_{t-1},x_{t}\right]+b_{o}\right)$$

(6) $$h_{t}=o_{t}*\tanh\left(C_{t}\right)$$

where $W_{o}$ and $b_{o}$ are the weight matrix and bias of the unit. We also add a single neuron to the last layer of LSTM for obtaining the predicted label.

#### 2.1.2. LSTM with Attention Mechanism

Although LSTM can protect and control the state of neurons by manipulating the input gate, output gate, and forget gate, solving the problems of traditional RNN neurons, it cannot provide a certain degree of interpretability for the importance of different input types of data. Here, we introduced the attention mechanism, a technology that allows the model to focus on important information and fully learn to absorb it [35]. Taking self-attention as an example, it contains three important matrices: Q, K, and V. Q is the query vector of the word, K is the “checked” vector, and V is the content vector. In this study, we introduced the attention mechanism after the last layer of LSTM model [36,37]. Suppose the embedding dimension of each hidden state is d, then $H\in R^{N\times d}$. We can obtain query, key, and value from the following projections:

(7) $$Q=H*W^{Q}$$

(8) $$K=H*W^{K}$$

(9) $$V=H*W^{V}$$

where $W^{Q},\;W^{K}$ and $W^{V}\in R^{d\times d}$ are the weight matrices.

We then calculate attention weight and attend the value to obtain the final output by the following equation, which enables judging the importance of different hidden states:

(10) $$H^{\prime }=Softmax\left(\frac{Q*K^{T}}{\sqrt{d}}\right)*V$$

which can judge the importance of different hidden states.

#### 2.1.3. RF

RF is a non-linear ensemble method based on decision trees. By introducing bootstrap aggregation (i.e., bagging), multiple decision trees can be integrated and are then combined to create a predictor based on the mean of each voting result from each decision tree [38]. RF is highly interpretable because the model is a tree-like diagram where each node has a partitioning rule.

### 2.2. Model Evaluation

The model accuracy is evaluated based on the predicted and observed weekly dengue cases by computing the root mean squared error (*RMSE*) and mean absolute error (*MAE*) as follows [39]:

(11) $$RMSE=\sqrt{\frac{1}{n}\sum {\left(o_{i}-y_{i}\right)}^{2}},$$

(12) $$MAE=\frac{1}{n}\sum_{i=1}^{n}\left|o_{i}-y_{i}\right|,$$

where $o_{i}$ represents the observed value for epi week i, and $y_{i}$ represents the predicted value for epi week i. The larger the indices’ values, the worse the model effect.

### 2.3. Multi-Date Ahead Prediction Scenarios

In this study, four prediction scenarios (i.e., 1- to 4-week-ahead) with two groups of input features (i.e., modeling with climate and environmental factors, and modeling with historical dengue data and climate and environmental factors) were used.

## 3. Experiments

To evaluate the performance of the proposed framework, we selected the Federal District of Brazil and Fortaleza as study sites. The Federal District of Brazil is the smallest federative unit without municipalities, and intense urban land expansion and population growth make dengue a major public health issue in recent years [40,41,42]. Moreover, Fortaleza is the fifth most densely populated city in Brazil and exhibits great socioeconomic inequality where intense dengue epidemics occurred in recent decades due to population concentration and urban inequality [25,41,43,44]. The experiments of city-level forecasting of weekly dengue cases were implemented as follows: (1) generating the time series of weekly dengue cases; (2) delineating the dengue transmission areas and generating the time series of risk predictors based on the GEE platform; (3) constructing, training and evaluating multi-date ahead and multi-scenario models. The experimental steps are detailed hereafter.

### 3.1. Generating the Time Series of Weekly Dengue Cases

We obtained the dengue cases data for the Federal District and Fortaleza from a publicly available dataset, namely Arboviral disease record data-Dengue and Chikungunya, Brazil [41], which collects the dengue notification cases submitted to the Notifiable Diseases Information System in Brazil (SINAN) from 2013 to 2020. We counted the number of dengue cases per epidemiological week and generated a time series of 418 weekly dengue cases per city. We then calculated the natural log-transformation for weekly dengue cases plus 1 to obtain a more stable time series.

### 3.2. Delineating the Dengue Transmission Areas and Generating the Time Series of Risk Predictors Based on the GEE Platform

**Figure 2:**
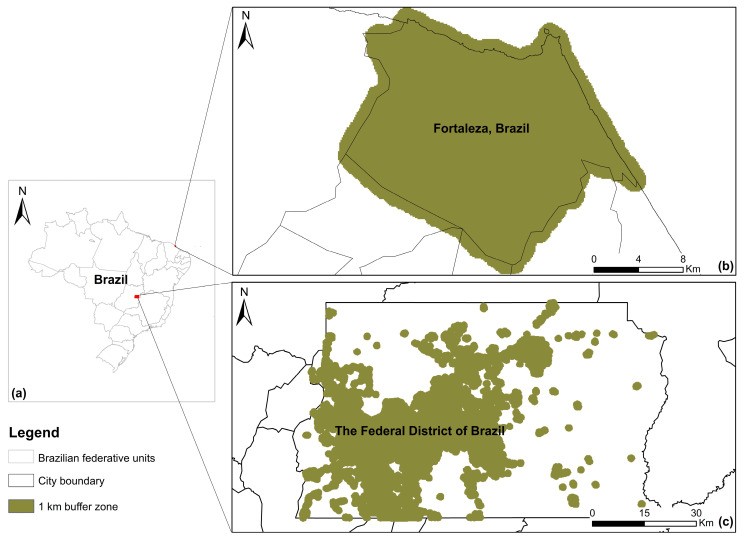
The geographical distribution of the study sites (**a**) and the main area of dengue transmission in the Federal District (**b**) and Fortaleza (**c**) in Brazil.

We used the impervious map of 2017 derived from the annual maps of global artificial impervious area (GAIA), a dataset containing the annual change in global impervious surface during 1985–2018 with 30 m spatial resolution [45]. We then defined a buffer zone of 1 km around the imperious surface to delimitate the predominant area of dengue transmission for each city (Figure 2). Due to no significant dynamic changes in the impervious surface during 2013–2020, we used the data of 2017 to characterize the predominant area of dengue transmission.

Four climate and environmental factors were used as risk predictors in this study, including total rainfall (R_sum_), mean temperature (T_mean_), mean relative humidity (RH_mean_), and mean normalized difference vegetation index (NDVI_mean_). To generate the time series with a weekly temporal resolution for each factor during 2013–2020, we first selected the data covering the predominant area of dengue transmission (Figure 2) with daily or sub-daily temporal resolution. R_sum_, T_mean_ and RH_mean_ were derived from a global, ready-to-use dataset of land surface states and fluxes, namely Global Land Data Assimilation System Version 2.1 (GLDAS-2.1) [46], and NDVI_mean_ was derived from MODIS MOD09GA dataset [47]. Then, all raster layers between the beginning date and the end date of each epidemiological week were selected, and a weekly composite was generated using a composition algorithm. Here, the sum was used to compute the total precipitation per pixel for each week, and the mean was used to compute the average status of temperature, relative humidity, and NDVI per pixel for each week. A total of 418 weekly composites were generated for each climate and environmental factor. Finally, for each weekly composite, we obtained a mean value by spatially aggregating the pixel values covering the buffer zone. A time series of 418 values was generated for each climate and environmental factor. Detailed information on big geospatial data used in this study is presented in Table 1.

### 3.3. Model Construction, Training, and Evaluation Using Google Colab

The time series of R_sum_, T_mean_, RH_mean_, NDVI_mean_, and naturally log-transformed weekly dengue cases were combined to generate the dataset for model training and evaluation. Datasets of the Federal District and Fortaleza were split respectively, taking data in the first 326 weeks for model training and the rest for model evaluation. To fully compare the performance of RF, LSTM, and LSTM-ATT, we considered time lags in advance of dengue epidemics and defined 1- to 4-week ahead prediction scenarios with two groups of input features (i.e., climate and environmental factors with and without historical dengue data. A total of 24 models were built. RF models were implemented using an open-source package scikit-learn [48] for Python 3.7, and LSTM and LSTM-ATT models were implemented based on Tensorflow 2.8.2 and Python 3.7. All experiments were run in Google Colab. RF models were trained using default parameters, and the parameters of LSTM and LSTM-ATT models are listed in Table 2.

## 4. Results

### 4.1. Time Series of Climate and Environmental Factors and Weekly Dengue Cases

**Figure 3:**
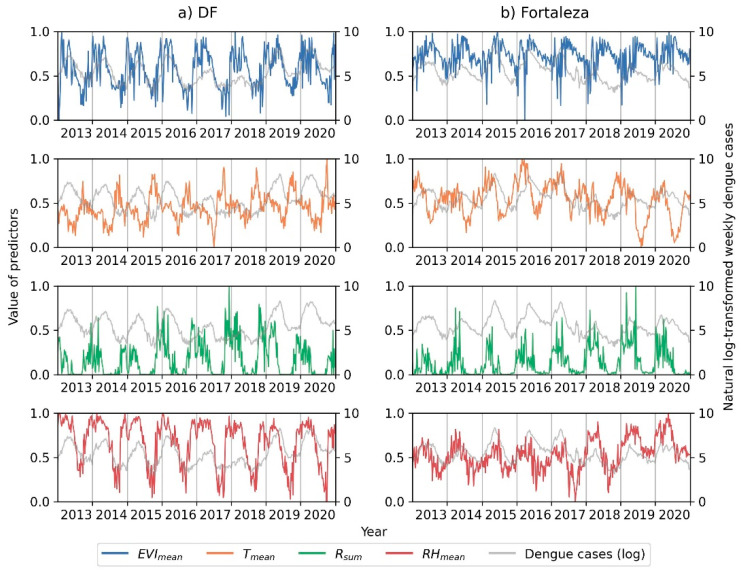
Illustration of the time series of climate and vegetation factors and natural log-transformed weekly dengue cases during 2013–2020 in the Federal District (**a**) and Fortaleza (**b**) in Brazil.

Figure 3 presents the temporal pattern of weekly dengue cases in the Federal District and Fortaleza in Brazil during 2013–2020. Dengue outbreaks could be observed each year for the two cities, and similar temporal patterns were observed, with the epidemic season mainly from February to May each year.

### 4.2. Outcomes of RF, LSTM and LSTM-ATT Modeling

**Figure 4:**
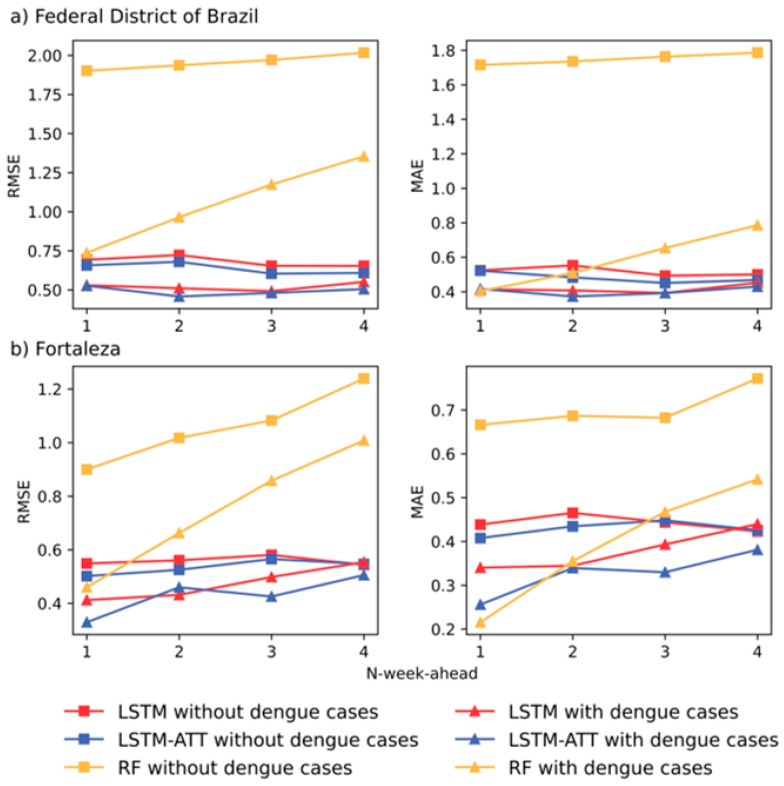
Accuracy comparison of multi-step ahead RF, LSTM and LSTM-ATT modeling with two groups of input features (i.e., with or without historical dengue data) using RMSE and MAE.

**Figure 5:**
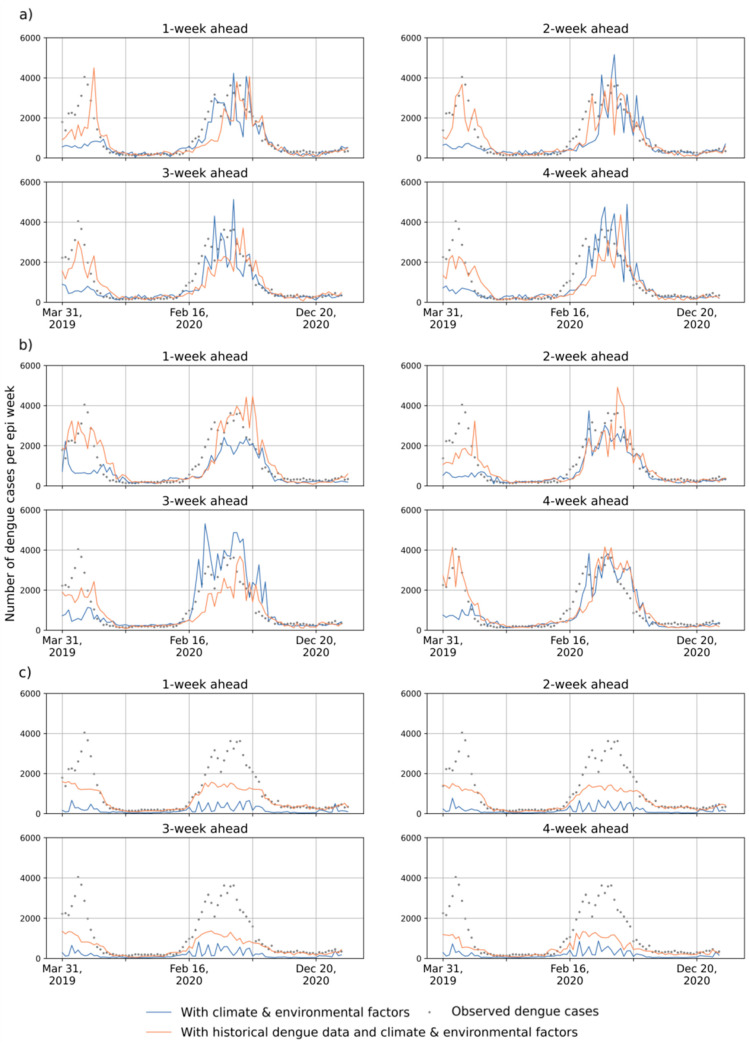
Illustration of the 1- to 4-week-ahead prediction with two groups of the input features for the dataset of the Federal District using (**a**) LSTM, (**b**) LSTM-ATT, and (**c**) RF, respectively. The grey points represent the number of observed cases per week. Orange curves represent the number of predicted cases per week with historical dengue data, climate factors, and vegetation factors. Blue curves represent the number of predicted cases per epi week with climate and vegetation factors.

**Figure 6:**
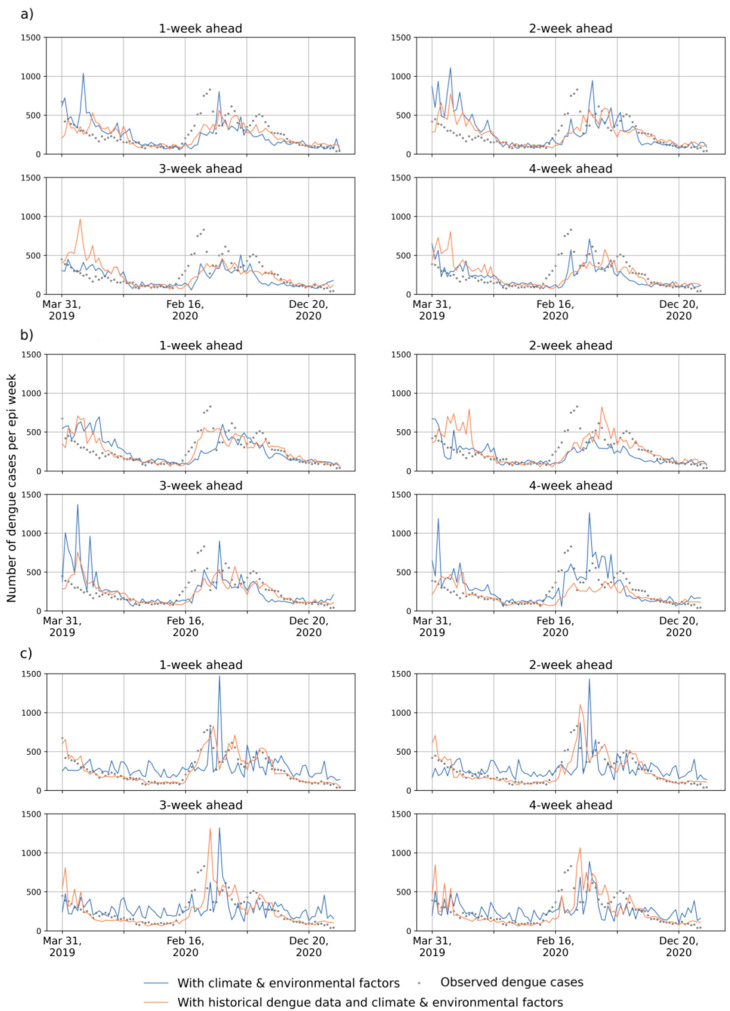
Illustration of the 1- to 4-week-ahead prediction with two groups of the input features for the Fortaleza using (**a**) LSTM, (**b**) LSTM-ATT, and (**c**) RF, respectively. The grey points represent the number of observed cases per epi week. Orange curves represent the number of predicted cases per epi week with historical dengue data, climate factors, and vegetation factors. Blue curves represent the number of predicted cases per epi week with climate and vegetation factors.

Figure 4 and Table A1 show the prediction accuracies in the Federal District and Fortaleza in Brazil. Figure 5 and Figure 6 present the predicted and observed curves of weekly dengue cases during 2019–2020 in the Federal District and Fortaleza, respectively. We can draw the following general conclusions:

1. RF models frequently have higher prediction errors than LSTM and LSTM-ATT models, and introducing historical dengue data as one of the input features can improve the performance of RF models for 1- to 4-week ahead predictions (see the yellow lines in Figure 4). Most of the predicted curves of RF models differed greatly from those of the observed cases, especially on the dataset of the Federal District of Brazil (Figure 5c).
2. All the blue and red lines are relatively stable in Figure 4, which suggests a slight difference in the accuracies of the 1- to 4-week ahead predictions for both LSTM and LSTM-ATT models.
3. The red curves with squares are frequently above the blue curves with squares in Figure 4, which suggests that LSTM modeling can benefit from an attention mechanism, which can further achieve performance lift in most cases. Similarly, the red curves with triangles are frequently above the blue curve with triangles in Figure 4, which suggests that LSTM-ATT modeling can also benefit from an attention mechanism.

The red curves with squares are frequently above the red curves with triangles in Figure 4, which suggests that LSTM modeling can benefit from using historical dengue data as one of the input features. Similar results could be observed for LSTM-ATT modeling, where the blue curves with squares are frequently above the blue curves with triangles. Moreover, the predicted curves are more stable compared to those of the predictions only with climate and environmental factors (Figure 5 and Figure 6).

## 5. Discussion

This study developed an efficient, accurate, and timely framework for city-level prediction of weekly dengue cases by integrating the GEE-based generation of risk predictors time series, historical dengue data, and Google Colab-based modeling. This study demonstrates the potential of multi-source geospatial data and cloud computing for the generation of dengue risk predictors and the power of Google Colab for developing various machine learning and deep learning models to predict dengue risk in advance.

The GEE platform allows assessing big geospatial data freely, avoids downloading and preprocessing multi-source data, and improves the efficiency and timeliness of generating the time series of dengue risk predictors using parallel data processing in the Google Cloud. A recent review of big geospatial data and data-driven models for dengue risk prediction shows that the GEE platform hosts global-scale satellite images and ready-to-use products on different topics that provide sufficient data sources to identify diverse driving factors at different spatial (e.g., health units, neighborhood, city, state/province, and country) and temporal scales (e.g., weekly and monthly) [49]. Additionally, a GEE-based web application has been developed to support malaria early warning by effectively generating environmental factors (e.g., daytime and nighttime land surface temperature, vegetation indices, and total precipitation) at the district scale in Ethiopia [16], which confirmed the effectiveness of the GEE platform in disease early warning.

We found that LSTM-ATT modeling with historical time series of weekly dengue cases and other driving factors frequently outperformed RF and LSTM models. Previous studies also confirmed that historical dengue data of a specific epidemic area provide the temporal characteristics of dengue transmission and help to improve the prediction accuracy of future dengue cases for the area and its neighboring areas [24,49,50]. In addition, a previous study forecasted the monthly dengue incidence rates for 20 provinces in Vietnam and confirmed that LSTM-ATT frequently outperformed other deep learning models [26]. Despite the good performance of LSTM and LSTM-ATT models in this study, RF has been used in several studies as it is easy to be implemented. Some facts limit the prediction accuracy of RF. It cannot quantify the relationships between the time series of dengue cases and risk predictors using a specific equation and may suffer from extrapolation problems, with predicted values being hard to be beyond the range of that in the training set [10]. That is to say, an underestimation of dengue cases can be observed while unprecedented outbreaks occur. By contrast, LSTM is capable of capturing the long-term dependency and non-linearity in the complex system of dengue transmission and permits adjusting the parameter time step to better quantify the impact of climate and environmental factors on dengue transmission; however, LSTM models lose information due to passing information across several sequence steps, and thus it will be worse in a long sequence. The problem can be mitigated by integrating LSTM with the attention mechanism, which enhances the power of information exploration by creating output for each sequence step. Besides, attention also provides a certain degree of interpretability for the importance of different hidden states [26].

The experiments in the Federal District and Fortaleza in Brazil confirmed the feasibility of the proposed framework to a certain extent; however, there are some limitations while using the proposed framework in real-world applications. For example, many climate and environmental factors could be considered in the dengue risk prediction based on the GEE platform; however, other important factors could not be quantified, such as the cycle of dengue genotype, population mobility, mosquito population, and immune status [51,52,53,54]. Especially, population mobility, an important factor influencing the spatial spread of dengue fever between geographical units (e.g., between cities or provinces), has been characterized using mobile phone data and public transportation data and used as one of the input features to improve the accuracy of dengue risk prediction in recent studies [25,52]. Future studies should consider how to explore big geospatial data to characterize the population mobility proxy and use it together with GEE-based climate and environmental factors as input features to model the future dengue risk. Moreover, other deep learning models dealing with time series risk prediction, such as GRU and Transformer, have been used in risk prediction of infectious diseases [26,55], which should be integrated into the proposed framework to understand the performance and time consumption. Lastly, this study solely adapted to the prediction of dengue cases 1 to 4 weeks in advance, which provides information on the temporal dynamics of dengue risk. Future studies should consider how to integrate more needs for dengue prevention and control into the proposed framework, such as predicting the peak intensity and peak timing of dengue epidemics and dengue outbreaks [25,56].

This study highlights the potential of the GEE platform and Google Colab in dengue risk prediction and also shows the benefits of using historical dengue data as one of the input features and attention mechanisms while LSTM modeling, which has important implications for future dengue risk prediction in terms of improving the effectiveness and accuracy of future dengue risk prediction. Early and accurate information on dengue transmission risk can guide the decision-making for dengue prevention and control and allow more time for the implementation of strategies.

## 6. Conclusions

Climate change, urbanization, and population growth highlight the importance of efficient and accurate dengue risk prediction. Multi-source data downloading and processing to identify dengue risk predictors and significant time and computational resources consumed to develop deep learning models locally make dengue risk prediction a challenge. This study used GEE-based geospatial data cloud computing to effectively generate the time series of climate and environmental factors and proposed accurate forecasting of weekly dengue cases based on LSTM modeling and an attention mechanism using Google Colab, considering total precipitation, mean temperature, mean relative humidity, mean NDVI, and historical dengue cases as input features. Our findings show the great potential of GEE-based geospatial data analysis and Google Colab-based deep learning modeling for facilitating dengue risk prediction for broader use in public health.

## Figures and Tables

**Table 1:** The big geospatial data used to delineate the main area of dengue transmission in cities and generate the time series of dengue risk predictors in this study.

Dengue Risk Predictors and Epidemiological Variables		Data Sources	Spatial Resolution	Temporal Resolution	Period
Climate	Precipitation per week (R_sum_)	GLDAS-2.1	27,000 m	3-hourly	2000 to present
	Mean temperature per week (T_mean_)				
	Mean relative humidity per week (RH_mean_)				
Environment	Mean NDVI per week (NDVI_mean_)	MOD09GA	500 m	Daily	2000 to present
Epidemiology	Number of dengue cases per week	Brazilian arboviral disease by [41]	City-level	Weekly	2013–2020
Dengue transmission areas	1 km buffer around the imperious surface in cities	GAIA	30 m	Annual	2017

**Table 2:** Summary of the parameters in LSTM and LSTM-ATT models. The time step denotes the length of the input features to make the prediction. The loss function quantifies the differences between the predicted value and the ground truth. The number of units is the number of neurons in the LSTM layer. The epoch is the number of completed training, while all data in the training set are used. Batch size represents the size of data in each batch for training the model. The learning rate refers to the step rate for updating parameters in backpropagation. Optimizer is the updating algorithm. Attention size is the embedding dimension of the weight matrix in the attention layer.

Parameters	LSTM without Dengue Cases	LSTM with Dengue Cases	LSTM-ATT without Dengue Cases	LSTM-ATT with Dengue Cases
Time step	12	12	12	12
Loss function	MSE	MSE	MSE	MSE
Number of units	64	64	64	64
Epoch	1000	1500	1500	2000
Batch size	12	12	12	12
Learning rate	0.005	0.003	0.005	0.003
Optimizer	Adam	Adam	Adam	Adam
Attention Size	-	-	64	64

## Appendix A

The data of weekly dengue cases and risk predictors and the codes for RF, LSTM and LSTM-ATT modeling are available online at https://github.com/geeigsnrr/Dengue-risk-forecasting-IJERPH (accessed on 9 October 2022).

## Appendix B

**Table A1:** The RMSE and MAE values of multi-step ahead RF, LSTM, and LSTM-ATT models with two groups of input features for the Federal District and Fortaleza in Brazil.

**Models**			**Federal District of Brazil**		**Fortaleza**	
			**RMSE**	**MAE**	**RMSE**	**MAE**
LSTM	LSTM without dengue cases	1-week	0.6929	0.5238	0.5488	0.4385
		2-week	0.7231	0.5527	0.5606	0.4652
		3-week	0.6540	0.4930	0.5808	0.4437
		4-week	0.6535	0.4999	0.5439	0.4229
	LSTM with dengue cases	1-week	0.5299	0.4137	0.4123	0.3403
		2-week	0.5105	0.4074	0.4317	0.3446
		3-week	0.4920	0.3931	0.4983	0.3932
		4-week	0.5509	0.4519	0.5539	0.4397
	LSTM-ATT without dengue cases	1-week	0.6570	0.5222	0.5017	0.4077
		2-week	0.6798	0.4827	0.5251	0.4345
		3-week	0.6037	0.4505	0.5653	0.4481
		4-week	0.6083	0.4677	0.5467	0.4260
	LSTM-ATT with dengue cases	1-week	0.5265	0.4162	0.3292	0.2560
		2-week	0.4579	0.3723	0.4598	0.3394
		3-week	0.4805	0.3920	0.4254	0.3298
		4-week	0.5049	0.4295	0.5053	0.3811
RF	RF without dengue cases	1-week	1.9010	1.7157	0.8998	0.6661
		2-week	1.9362	1.7358	1.0179	0.6866
		3-week	1.9702	1.7637	1.0827	0.6824
		4-week	2.0166	1.7864	1.2393	0.7717
	RF with dengue cases	1-week	0.7371	0.4041	0.4601	0.2156
		2-week	0.9646	0.5087	0.6626	0.3550
		3-week	1.1738	0.6531	0.8581	0.4675
		4-week	1.3540	0.7855	1.0079	0.5417
